# The therapeutic effects of berberine plus sitagliptin in a rat model of fatty liver disease 

**DOI:** 10.22038/ijbms.2021.52239.11822

**Published:** 2021-04

**Authors:** Soraya Mehrdoost, Parichehreh Yaghmaei, Hanieh Jafary, Azadeh Ebrahim-Habibi

**Affiliations:** 1 Department of Biology, Faculty of Basic Sciences, Science and Research Branch, Islamic Azad University, Tehran, Iran; 2 Biosensor Research Center, Endocrinology and Metabolism Molecular-Cellular Sciences Institute, Tehran University of Medical Sciences, Tehran, Iran; 3 Endocrinology and Metabolism Research Center, Endocrinology and Metabolism Clinical Sciences Institute, Tehran University of Medical Sciences, Tehran, Iran

**Keywords:** Adiponectin receptor2, DDP-4, Glucose ttransporter type 4, Natural compound, Non-alcoholic fatty liver - disease, Pho-ERK/ERK

## Abstract

**Objective(s):**

Fatty liver disease (FLD) is a disorder related to accumulation of excess fat within the hepatocytes. In this study, the effects of Berberine, a natural compound, and Sitagliptin as a DPP-4 inhibitor, were observed in a rat model of FLD.

**Materials and Methods:**

Forty male rats were divided into five groups (n=6) including the control group (normal food and water), high-fat group (high-fat diet (HF) for 6 weeks), Berberine group (HF with oral administration of Berberine at 150 mg/kg for 6 weeks), Sitagliptin group (HF with oral administration of Sitagliptin at 10 mg/kg for 6 weeks), and Berberine/ Sitagliptin group (HF diet within combination with oral administration of Berberine 75 mg/kg and Sitagliptin 5 mg/kg for 6 weeks). Animals were examined for weight gain, serum and hepatic biochemical parameters, tissue histology, expression of glucose transporter type 4 (GLUT4) mRNA, and protein expression of Adiponectin receptor2 (AdipoR2) and extracellular signal-regulated kinase (ERK) and phoERK.

**Results:**

The results showed that ALT, AST, lipid profile, insulin, glucose, MDA, and TNF-α were significantly improved in high-fat rats treated with Berberine/ Sitagliptin compared with HF and Sitagliptin, and Berberine alone groups. SOD and adiponectin levels in Berberine/ Sitagliptin group were also significantly increased compared with the other groups. Immunoblot analysis showed that the expression of pho-ERK/ERK was significantly decreased and expression of AdipoR2 significantly increased in the Berberine/ Sitagliptin group compared with other groups.

**Conclusion:**

Co-administration of Berberine and Sitagliptin is an effective therapeutic regimen for conditions associated with hyperlipidemia.

## Introduction

Non-alcoholic fatty liver disease (NAFLD) is one of the common disorders leading to fat accumulation within the hepatocytes, increased liver enzymes, and obesity (1). Expansion of adipose tissue in obesity reduces its ability to store excess energy, resulting in increased fat dysfunction and insulin resistance, leading to lipolysis. Circulating free fatty acids (FFAs) increase and adiponectin decreases, resulting in intrahepatic fat accumulation, which increases lipogenesis. If adipose tissue expands, immune cells that produce cytokines and interleukins penetrate the tissue. When obesity is not successfully controlled in the simple steatosis stage, immune cells also invade the liver and play an important role in the process of low-grade but chronic intrahepatic inflammation. When inflammation is prolonged, fibrogenesis begins with the involvement of hepatic stellate cells. Intensification of fibrogenesis leads to cirrhosis, which eventually leads to scar tissue, apoptosis, and cell death (2). Although NAFLD is clinically asymptomatic and silent, it can cause inflammation, destruction of liver macrophages or Kupffer’s cells, and liver injury (3). The most clinical importance of this disease is when the disease is diagnosed with delay. The delayed diagnosis of the disease results in progress to non-alcoholic steatohepatitis (NASH) with its sequelae of destruction of Kupffer’s cells cirrhosis and liver cancer such as hepatocellular carcinoma (4). Fatty liver diseases have been reported with some metabolic syndromes such as hyperlipidemia, obesity, and type 2 diabetes (5). Insulin plays a role in the metabolism of fats and the imbalance in fat homeostasis induced by insulin deficiency results in triglyceride accumulation in the liver (6).

Up to 70% of patients with type II diabetes mellitus (T2DM) may have concurrent NAFLD. It has been demonstrated that these patients are at a higher risk of progression to NASH and advanced fibrosis (7). Based on our knowledge, appropriate treatment available for NASH is not yet known. Many hepatologists have tried to control NASH by changing lifestyle, weight loss, exercise, and standard therapeutic interventions to prevent concomitant diseases such as hyperlipidemia and T2DM (8, 9).

Dipeptidyl peptidase 4 (DPP- 4), also known as CD26 is one of the main molecules involved in the pathology of metabolic syndrome such as T2DM (10). DPP-4 is expressed in many cells (11), especially in skeletal muscles, liver, and adipose tissue, exerting pleiotropic effects via its peptidase activity (12). Since DDP-4 is highly expressed in hepatocytes it is involved in chronic liver diseases such as NAFLD, even prior to the development of diabetes (13). Indeed, DPP-4 is responsible for the rapid degradation of intestinal hormones such as glucagon-like peptide 1 (GLP-1) and gastric inhibitory polypeptide (GIP) (14). Chemical inhibition of DPP-4 activity or inactivation of DPP-4 genetically leads to increased levels of biological activity of GLP1 and GLP (15). DPP-4 inhibitors have been shown to improve insulin sensitivity resulting in improved liver lipid metabolism (16). Four DPP-4 inhibitors (sitagliptin, saxagliptin, linagliptin, and alogliptin) are currently available in the United States and many other countries (17). Herbal medicines are emerging as potential therapeutics for treatment of NAFLD. Berberine is an herbal compound with several medicinal activities. It has been recently shown that efficacy of Berberine against T2MD is due to its antioxidant and anti-inflammatory activities (18).

Oxidative stress is an inducer of mitogen-activated protein kinase (MAPK) and expression of pro-inflammatory genes. ERK is a known MAPK that has been shown to play a role in the fatty liver. ERK phosphorylation induces expression of Interleukin-8, intercellular adhesion molecule 1 (ICAM-1), and the CC motif of chemokine 5 ligand (CCL5; RANTES). Expression of these pre-inflammatory genes is associated with hepatitis in NAFLD (19).

Adiponectin is a circulating hormone derived from fat cells that regulates insulin sensitivity. Expression of adiponectin receptors has been shown to be closely related to insulin resistance. AdipoR1 is expressed in most tissues, including skeletal muscle, while AdipoR2 is mostly expressed in the liver (20). There is a debate about the expression of adiponectin receptors in animal models of obesity (21). Intensive research efforts are needed to evaluate the expression and role of various adiponectin receptors in liver disease. In this regard, we examined Adipo2 expression in the liver.

GLUT-4 has recently received especial attention in the field of diabetes and insulin resistance, and it has been reported that changes in its hepatic expression affect glucose homeostasis (22). Due to insulin resistance, GLUT-4 signaling is impaired in several tissues, including the liver. Therefore, its expression in relation to non-alcoholic fatty liver seems necessary (23). Expression of this receptor is also reduced in the liver of diabetic rats (24).

The effect of co-administration of Sitagliptin and Berberine on biochemical and histological parameters, insulin resistance as well as GLUT4 gene expression in NAFLD has not yet been investigated. Here, we have tested the hypothesis whether combination of Sitagliptin, as a DPP-4 inhibitor and Berberine, as an antioxidant and anti-inflammatory compound, can reduce fatty liver and improve liver function through AdipoR2 and ERK and GLUT4 signaling pathways.

## Materials and Methods

***Preparation of fat emulsion***

The high-fat emulsion diet was composed of 77% fat, 14% milk powder, and 9% carbohydrates. Details of these compounds are shown in Table 1. In this emulsion, protein, carbohydrates, and fat were provided from total milk powder, saccharose, and corn oil, respectively. Each diet was supplemented with a vitamin and mineral mixture. This emulsion was stored at 4 °C, warmed in a 42 °C water bath, and fully mixed before use (25).

***Animals and treatment***

A total of 40 male Sprague–Dawley rats (254±20 g) were supplied by the Experimental Animal Center, Razi Vaccine and Serum Research Institute. This study was performed in accordance with the published guideline of the Care and Use of Laboratory Animals (NIH Publication, 8th edition, revised 2011). The study protocol was also approved by the local Animal Care Committee (clearance code: IR.IAU.SRB.REC.1398.035). The fatty liver was induced in rats as was previously described by Zou *et al* (25).

40 rats were randomly divided into five equal groups each of 8 rats, the control group (received normal food and water), the high-fat group (received daily high-fat foods 10 ml/kg/day for 6 weeks), the Berberine group (received daily high-fat foods with oral administration of Berberine at a dose of 150 mg/kg for 6 weeks), the Sitagliptin group (received daily high-fat foods with oral administration of Sitagliptin at a dose of 10 mg/kg for 6 weeks), and the Berberine/ Sitagliptin group (received daily high-fat foods in combination with oral administration of Berberine 75 mg/kg and Sitagliptin 5 mg/kg for 6 weeks)

At the end of the test and 14 hours after fasting Rats were sacrificed under anesthesia. Blood sampling was performed using a 5 ml syringe from the heart ventricle. The livers were immediately removed and weighed.

***Analytical procedures***

The serum concentration of aspartate aminotransferase (AST), alanine aminotransferase (ALT), total cholesterol (TC), high-density lipoprotein-C (HDL-C), low-density lipoprotein-C (LDL-C), triglyceride (TG), and glucose of each rat was measured by a biochemical autoanalyzer (SelectraProM, France). The levels of serum-FFAs, serum insulin, adiponectin, and tumor necrosis factor α (TNF-α), along with liver homogenate malondialdehyde (MDA) and liver superoxide dismutase (SOD) were measured by commercial Elisa kits (Navand Salamat, Iran). Hepatic concentrations of TC and TG were also measured using Floch’s method (25). In brief, liver tissue was dissected and 50 mg of the tissue was placed in chloroform/methanol (2:1 by volume) and mixed. TG and TC content in the extracts were measured using commercial kits from Wako Chemicals (Richmond, VA). 

***Histological evaluation***

To confirm the induction of fatty liver, the liver tissues were immediately fixed in 10% buffered formalin, paraffin-embedded, and stained with hematoxylin-eosin. Fatty change was assessed according to the percentage of hepatocytes containing macro-vesicles (25).

***Western blotting***

The collected tissue (0.05–0.3 g of each tissue) was homogenized in 1.0 mL of the Ripa buffer containing (100 mM Trizma, 10 mM EDTA, 1% sodium dodecyl sulfate(SDS), 100 mM sodium fluoride, 10 mM sodium pyrophosphate, 10 mM sodium orthovanadate, 2 mM phenylmethylsulfonyl fluoride (PMSF), and 0.25 mg aprotinin protease inhibitor) to obtain the tissue lysates. The lysates were incubated with 10% Triton X-100 for 30 min and then centrifuged at 14,000 rpm for 20 min at 4 °C. The supernatants were collected, and protein concentrations were determined using a commercial BCA kit (Abcam, USA) using bovine serum albumin (BSA) as a reference standard. The protein samples were treated with the Laemmli sample buffer and boiled for 5 min before loading onto a 12% SDS-PAGE gel (30 μg protein). Electro transfer of the proteins from the gel to polyvinylidene fluoride (PVDF) membrane was then performed for 12 hr at 50 V. Non-specific proteins binding (NSPB) on the PVDF membrane were blocked by a 2-hr preincubation in blocking buffer (1% BSA, 10 mM Tris, 150 mM NaCl and 0.02% Tween 20). For immune detection, the PVDF membranes were incubated overnight at 4 °C with antibodies against AdipoR2 (Boster Biological Technology, Pleasanton CA, USA) and ERK (Boster Biological Technology, Pleasanton CA, USA) and pho-ERK (Boster Biological Technology, Pleasanton CA, USA), or β-actin (Boster Biological Technology, Pleasanton CA, USA). The membranes were then incubated with a secondary antibody (Sigma Aldrich, USA) and afterward with enhanced chemiluminescence (ECL) (Thermo Fisher Scientific) for 1–2 min. β-actin was used to normalize protein expression. The density of protein bands was determined using the ImageJ Version 1.44 software package (NIH, USA), and then the percentage area under the curve of each band was divided by the percentage area under the curve of its corresponding actin band. The calculated values were then compared between groups as described previously (26).

***RNA extraction***

Total RNA was extracted with an RNA purification kit (Sinnaclon, Iran) in accordance with the manufacturer’s protocol and the extracted RNAs were qualified with NanoDrop (Nanodrop Technologies, USA). For GLUT4mRNA expression analysis, the extracted RNA was reverse-transcribed to cDNA using a cDNA Synthesis Kit (Sinnaclon, Iran).

***Real-time PCR***

The relative expression of the GLUT4 gene was measured by an ABI Prism 7300 sequence detection system (Applied Biosystems, USA) using SYBR green dye. The real-time PCR cycle consisted of 95 °C for 5 min as initial denaturation step followed by 40 cycles of amplification (95 °C for 15 sec, 60 °C for 1 min and 95 °C for 15 sec) and culminating with melt curve analysis. GAPDH was used as internal control, and each sample was repeated in triplicates. The normalized results with GAPDH were expressed as the fold changes and analyzed using the 2-ΔΔCT method (27). The following rat primer sequence sets were used: GLUT4 forward, 5’- TCACTATGCTCTGGTCTCTGTCTG, and reverse, 5’- GCTTTGATCCTTCCGAGTTTGTCC; and GAPDH forward, 5’- TGCCGCCTGGAGAAACC, and reverse, 5’- TGAAGTCGCAGGAGACAACC.

***Statistical analysis***

The findings are expressed as mean±standard deviation (SD). After analyzing the normal distribution of data and homogeneity of variances, one-way ANOVA was used to assess the statistical significance between groups followed by *post hoc* Tukey’s test. Data were analyzed with GraphPad InStat software package (ver. 2.02). Values of *P*≤0.05 were considered statistically significant. In histograms, the statistical difference between groups is shown with *P<0.05, **P<0.01, and ***P<0.001.

## Results

***Changes in body and liver weight***

**Figure 1:**
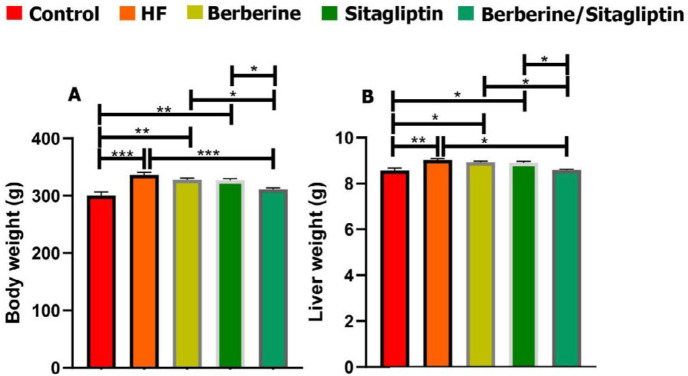
Effects of high fat on body (A) and liver (B) weight. Data are expressed as mean±SD. *P<0.05, **P<0.01, ***P<0.001 (one-way ANOVA with Tukey’s *post hoc* test)

The NAFLD model had been induced by feeding rats with high-fat emulsion (10 ml/kg/day) for 6 weeks, and the general condition of the rats remained good. The body and liver weight of all rats are presented in Figures 1A-B. 6 weeks after feeding, both body weight and liver weight in the high fat (HF) group were significantly greater than those of the other groups (Figures 1A and B). Combination of Berberine and Sitagliptin group showed lower body and liver weight compared with the HF group at 6 weeks post-treatment (Figure 1).

***Serum biochemical findings***

**Figure 2:**
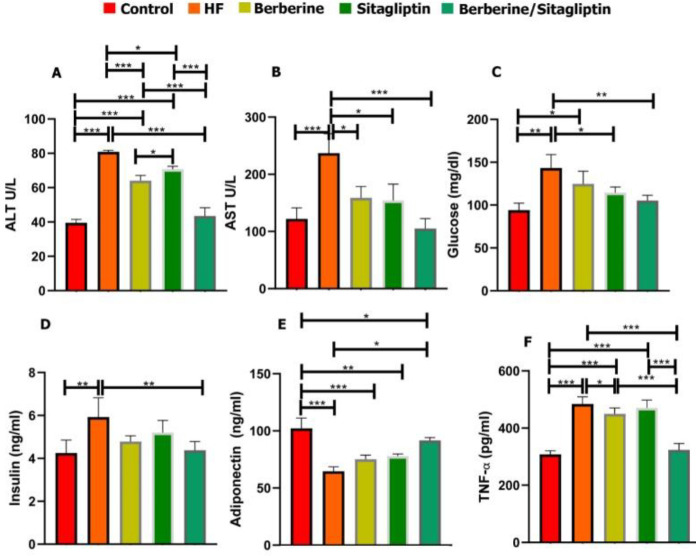
Serum content of ALT, AST, Glucose, Insulin, TNF-α, and adiponectin in control, HF, Berberine, Sitagliptin, and Berberine/ Sitagliptin treated groups. Serum ALT (A), AST(B), Glucose(C), Insulin (D), adiponectin (E), TNF-α (F) levels in Control, HF, Berberine, Sitagliptin, and Berberine/Sitagliptin treated groups. Data are expressed as mean±SD *P<0.05, **P<0.01, ***P<0.001 (one-way ANOVA with Tukey’s *post hoc* test)

**Figure 3:**
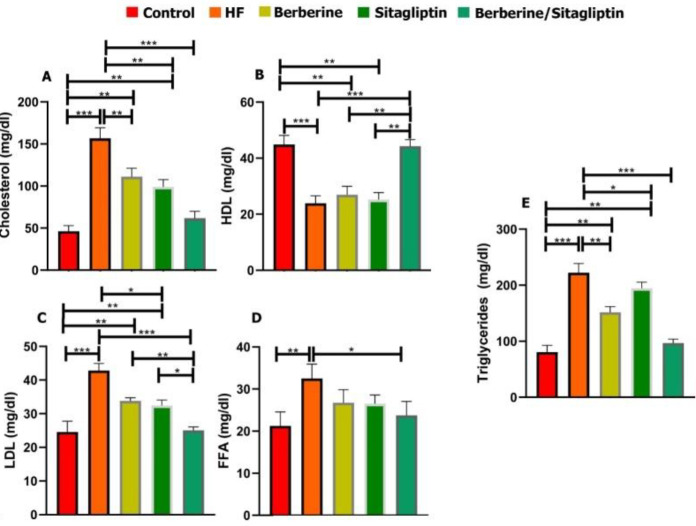
Serum content of Cholesterol, Triglyceride, FFA, HDL, LDL in control, HF, Berberine, Sitagliptin, and Berberine/ Sitagliptin treated groups. Cholesterol (A), HDL (B), LDL (C), FFA (D), and Triglyceride (E) levels in control, HF, Berberine, Sitagliptin, and Berberine/ Sitagliptin treated groups. Data are expressed as mean±SD. *P<0.05, **P<0.01, ***P<0.001 (one-way ANOVA with Tukey’s post hoc test)

FFA: free fatty acids; HDL: high-density lipoprotein; LDL: low-density lipoprotein; HF: high fat

**Figure 4:**
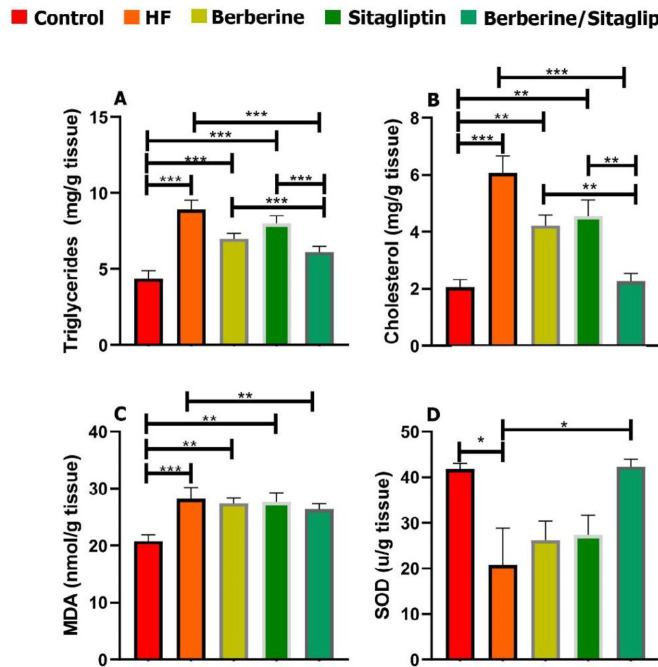
Effect of Berberine, Sitagliptin, and Berberine/ Sitagliptin on the hepatic triglyceride, cholesterol, MDA, and SOD of rats with HF diet. After 6 weeks of administration, combination of Berberine/ Sitagliptin significantly reduced hepatic triglyceride (A), cholesterol (B), and MDA (C) levels in rats with HF. SOD contents were significantly increased in the liver of rats with HF treated Berberine/ Sitagliptin (D). Data are expressed as mean±SD. *P<0.05, **P<0.01, ***P<0.001 (one-way ANOVA with Tukey’s *post hoc* test)

MDA: malondialdehyde; SOD: superoxide dismutase; Hf: HF: high fat

Serum levels of AST, ALT, TC, TG, FFA, LDL, glucose, insulin, and TNF-α were significantly higher in the HF group than those of the control, the Berberine, and Sitagliptin alone groups (*P<0.05) whereas the serum levels of HDL and adiponectin were lower in the HF group than those of other groups at 6 weeks post-treatment (Figures 2A-F, Figures 3A-E). Combination of Berberine and Sitagliptin could significantly increase the adiponectin levels of rats fed with HF diet compared with the HF group at 6 weeks post-treatment. The hepatic contents of TC, TG, and MDA were significantly increased in the HF group compared with the other groups (Figures 4A-C). The SOD activity was significantly decreased in the HF group compared with other groups (Figure 4D). In addition, a combination of Sitagliptin and Berberine simultaneously reduces the amount of TC, TG, and MDA in the liver tissues of rats fed with HF diet compared with the HF group at 6 weeks post-treatment (Figures 4A-C).

***Histological findings***

**Figure 5:**
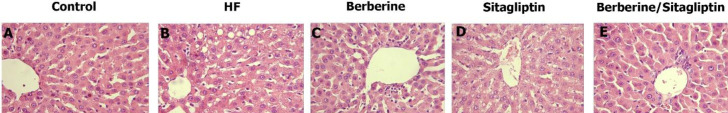
High-fat emulsion diet led to steatohepatitis. The liver of rats in control (A), HF (B), Berberine(C), Sitagliptin(D), and Berberine/ Sitagliptin(E) groups (H&E stain)

A diffuse and mild fatty change, indicated with intra-cytoplasmic vascular degeneration leading to acentric nuclei, was detected in the high-fat and Berberine and Sitagliptin alone groups, respectively (Figures 5A-D). There was no fatty change in the combination treatment group (Figure 5E).

***Western blotting***

**Figure 6:**
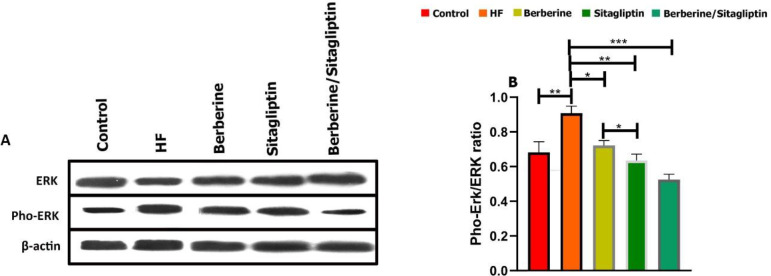
Western blots for ERK and Pho-ERK in rat liver tissue lysate. Western blot images (A) and relative expression of Proteins (B). The percentage area under the curve of each protein band was divided by the percentage area under the curve of the corresponding β-actin band, and the normalized data were statistically compared between groups. Data are expressed as mean±SD. (three independent experiments were performed in triplicate). *P<0.05. **P<0.01, ***P<0.001. (one-way ANOVA with Tukey’s *post hoc* test)

The relative expression levels of ERK and pho-ERK in liver tissue were assayed using immunoblotting. Western blotting revealed that the levels of phosphorylation of ERK were significantly up-regulated in the HF group compared with the other groups (Figure 6A). However, the phosphorylation level of ERK in the mitogen-activated protein kinase signaling pathway was significantly down-regulated by Berberine/ Sitagliptin compared with the HF diet group in the liver tissue (*P*<0.001) Also, phosphorylation level ERK by Berberine and Sitagliptin alone was significantly down-regulated compared with the HF diet group in the liver tissue (*P*<0.05, *P*<0.01, respectively) (Figure 6B).

**Figure 7:**
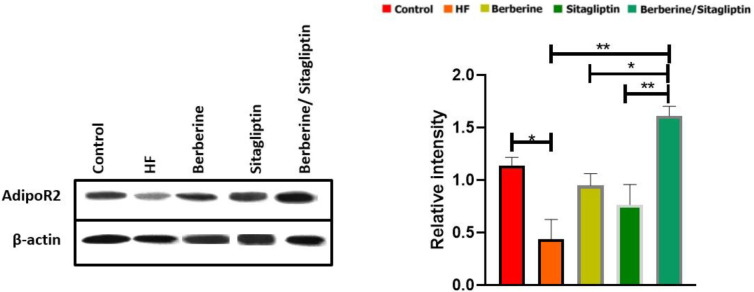
Western blots for AdipoR2 expression in liver tissue. The percentage area under the curve of each protein band was divided by the percentage area under the curve of the corresponding β-actin band, and the normalized data were statistically compared between groups. Data are expressed as mean±SD. (three independent experiments were performed in triplicate). *P<0.05. **P<0.01, ***P<0.001. (one-way ANOVA with Tukey’s *post hoc* test)

We then investigated protein level changes of AdipoR2 in the liver. Berberine/ Sitagliptin significantly increased protein levels of AdipoR2 in the liver compared with the HF group (*P*<0.01) and Berberine and Sitagliptin alone groups (*P*<0.01, *P*<0.05*, *respectively) (Figure 7).

***mRNA expression of GLUT4 in liver tissue***

**Figure 8:**
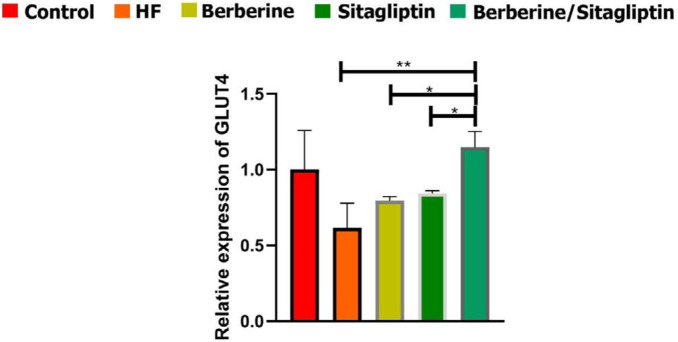
GLUT4 gene expression by real-time PCR. GLUCT4 gene expression level after normalization to β-actin in each group. *P< 0.05, **P< 0.01, (one-way ANOVA with Tukey’s *post hoc*)

The mRNA expression level of GLUT4 was evaluated by real-time PCR (Figure 8). GLUT4 was significantly down-regulated in the HF group compared with the other groups. The data also showed that combination of Berberine/ Sitagliptin significantly up-regulated mRNA expression level of GLUT4 compared with the HF group) *P*<0.01 (and Berberine and Sitagliptin alone treated groups) *P*<0.05( (Figure 6).

**Table 1:** Composition and caloric content of the high-fat emulsion diet (10 ml/kg/day) ingested via gavage in NASH model rats

**Component**	**High-fat emulsion**
**Corn oil (g) ** **Saccharose (g) ** **Total milk powder (g) ** **Cholesterol (g) ** **Sodium deoxycholate (g) ** **Tween 80 (g) ** **Propylene glycol (g) ** **Vitamin mixture (g) ** **Cooking salt (g) ** **Mineral mixture (g) ** **Distilled water (ml) ** **Total energy (kcal/l) **	400 150 80 100 10 36.4 31.1 2.5 10 1.5 300 4342

ALT: alanine aminotransferase; AST: aspartate aminotransferase

HF: high fat

ERK: extracellular signal-regulated kinase

## Discussion

High-fat diets (HFD) often cause oxidative stress, which can lead to various liver damages and NAFLD (25). In this study, oral administration of HFD induced oxidative stress, lipid peroxidation, steatosis, and increased ALT and AST activities in rats, confirming the successful formation of an animal model of NAFLD. Previous studies have shown that dietary fat intake causes weight gain (25), and findings of this study also showed that HFD increased body weight in the HF group, while treatment with a combination of Sitagliptin and Berberine resulted in treated groups showing no significant difference with the control group. Berberine has a significant effect on carbohydrate and fat metabolism. AMPK (adenosine monophosphate-activated protein kinase) is the main sensor of cell energy and a key regulator of lipid and glucose metabolism, and findings show that Berberine is able to activate the AMPK signaling pathway (28). Hormone-based therapies including GLP-1 and GIP agonists and dipeptidyl peptidase-4 (DPP-4) inhibitors have been linked to reduced obesity. Sitagliptin as a DPP-4 inhibitor increases the biological activity of incretin hormones (29) and may have a synergistic effect with Berberine, so that co-administration of these compounds may be an effective option for inducing weight loss.

Previous studies have shown that increasing the intake of HFD is involved in developing insulin resistance (25). Dpp4 inhibitors increase the biological activity of incretin hormones, thereby increasing the half-life of insulin and improving insulin resistance (30). After insulin stimulation, GLUT4 is distributed in the plasma membrane. In insulin resistance, a defect in this process reduces GLUT4 expression and displacement in major insulin target organs such as the liver and muscles (31). In the cultured human hepatocytes, Berberine increases Insulin receptor (InsR) mRNA and also activates AMPK in adipocytes, which has been shown to reduce insulin resistance. InsR in muscle also is regulated by Berberine, but mostly, hepatocytes have been studied because hepatic insulin resistance is an important driving force in hyperglycemia and type 2 diabetes (32). The synergistic effect of co-administration of Berberine/ Sitagliptin may have a better therapeutic effect, reducing hyperinsulinemia and fasting blood sugar, and improving insulin sensitivity in rats, suggesting a role in improving insulin resistance in NAFLD. Improving insulin resistance is an important approach to treating fatty liver. 

Insulin resistance reduces the inhibitory effect of insulin on lipolysis thus increasing the production of FFAs. An increase in hormone-sensitive lipase activity leads to increased triglyceride lipolysis and FFA flow to the liver (33, 34). Plasma FFA levels are associated with lipolysis and insulin resistance, as well as elevated TG and LDL levels. Previous studies have shown that Berberine induces LDL receptor (LDLR) expression and regulates plasma LDL homeostasis. An increase in hepatic LDLR expression leads to plasma clearance of LDL via its receptor-mediated endocytosis (32). Sitagliptin can increase the pre-insulin function of GLP-1 and improve insulin resistance, thus reducing fatty acid hydrolysis, so, Sitagliptin improves the levels of incretin hormones, thereby affecting fat metabolism (35). In our study, a high-fat emulsion diet reduced serum HDL levels. FFA, TC, TG, and LDL-C increased but the level of these parameters decreased in the treated groups, especially the Berberine/ Sitagliptin group. This suggests that Berberine and Sitagliptin may reduce blood lipids produced by insulin resistance through different and synergistic pathways.

Elevated serum levels of ALT and AST occur in NAFLD patients. Adiponectin decreases and TNFα increases in NAFLD and these two events are associated with insulin resistance (9). Adiponectin levels are inversely related to obesity and obesity-related complications. Adiponectin has been shown to increase insulin sensitivity in skeletal muscles and liver tissue .(36) Adiponectin increases peroxisome proliferator-activated receptor-a (PPAR-a) ligand activity and stimulates fatty acid oxidation and glucose uptake(37) Sitagliptin improves adipose tissue inflammation, metabolic syndrome, and fatty liver by regulating adiponectin and AMPK levels in obese rats, also, increased GLP-1 levels cause insulin-independent effects on adipose tissue and liver (36). Berberine counteracts insulin resistance by activating two proteins, sirtuin 1 (SIRT1), an acetylase, and AMPK, as a result of activation of SIRT-1 and AMPK pathways, adiponectin concentration increases, and increasing adiponectin is associated with regulation of β oxidation and glucose metabolism (38). Sitagliptin plays a role in reducing inflammatory factors such as TNFα by increasing adiponectin, antioxidant effect, and protective liver adipokine. Increased GLP-1 by Sitagliptin could be a possible mechanism of its antioxidant effect (39). Previous studies confirm the anti-inflammatory effects of Berberine, and treatment with it reduces pro-inflammatory factors, including TNF-α (18), also, Berberine reduces the expression of pro-inflammatory genes in an Ampk-dependent manner (40). In this study, administration of HFD increased ALT, AST, and TNFa and decreased adiponectin levels. The anti-inflammatory effects and improvement of insulin resistance in the treatment with Sitagliptin and Berberine-induced synergistic pathways may modulate hepatic transaminases (ALT AST), increase adiponectin, and reduce TNFα.

Increased production of Reactive oxygen species (ROS) is observed in animal models of fatty liver (25). SOD is essential for prevention of oxidative stress and inhibition of ROS under physiological conditions (41). Continuous exposure to ROS greatly leads to significant damage to cell structure and function. Free radicals react with lipids to produce hydroperoxides and endoperoxides that may be broken down to form reactants such as MDA that cause irreversible reactions with proteins, DNA, and phospholipids and lead to cell death (42). Studies have shown that Sitagliptin reduces oxidative stress and inflammation (39). Berberine up-regulates SOD mRNA expression in diabetic mice and has also been reported to increase expression levels of SIRT1, with multiple biological and antioxidant activities (18). In the HF group of this study, SOD activity decreased and MDA increased but in the Berberine/ Sitagliptin treatment group, no significant difference was observed with the control group. This finding indicates that treatment with Berberine/ Sitagliptin is more effective in reducing oxidative stress and treatment of HFD-induced lipid peroxidation than treatment with each compound alone.

When there is an imbalance between energy intake and energy consumption, or when there is an inherent problem in storing excess energy as lipid (triacylglycerol) in adipose tissue stores, lipid accumulation occurs in other organs of the body that are not designed to accumulate fat, such as the liver. Increased FFA flow from adipose tissue increases its accumulation in liver tissue (43). The liver plays an important role in lipid metabolism, and Berberine exerts important effects on the regulation of lipid metabolism by regulating intranuclear transcription factors, including Forkhead transcription factor O1 (FoxO1), and sterol regulatory element-binding protein 1c (SREBP1). Berberine reduces the expression of these factors as well as the expression of fatty acid synthase; on the other hand, it possibly improves metabolic activity by modulating the expression of PPARs in the liver (44). Decreased hepatic AMPK activity reduces the phosphorylation of acetyl coenzyme A carboxylates (ACC) thereby reducing fatty acid oxidation and increasing lipogenesis(32). High expression of AMPK in the liver reduces triglyceride content and lipogenesis in type 2 diabetic rats. AMPK can also regulate fatty acid metabolism by regulating mitochondrial biogenesis(45). Berberine increases AMPK activity (32). Sitagliptin can increase the pre insulin function of GLP-1 and reduce fatty acid hydrolysis by improving insulin resistance. Studies have shown that Sitagliptin can affect fatty liver induced by HFD via reducing intrahepatic lipogenesis and increasing lipid oxidation (35). In the present study, co-administration of Berberine/ Sitagliptin showed stronger effects on lowering hepatic cholesterol and triglycerides than when administered alone.

Inhibition of ERK inhibits pro-inflammatory gene expression (19). Previous studies have shown that p ERK activity in liver stellate cells may be linked to the development of hepatic steatosis, suggesting a role in the development of hepatic steatosis (46). According to previous studies, Sitagliptin can activate the AMPK signaling pathway, inhibit the MAPK signaling pathway and reduce ERK phosphorylation, which plays a key role in inducing the anti-inflammatory effects of this drug (47). Berberine has been reported to be able to inactivate ERK to reduce p ERK-induced inflammation (48). In line with these results, we have shown that co-administration of Berberine/Sitagliptin shows stronger effects in reducing hepatic ERK phosphorylation and possibly through this effect reduces hepatitis and improves fatty liver. 

Adiponectin must bind to its receptors to have beneficial biological effects such as insulin-sensitizing (49). Adiponectin inhibits hepatocellular carcinoma cell proliferation by blocking downstream pathways including signal transducer and activator of transcription-3 (STAT-3), protein kinase B (AKT), and mammalian target of rapamycin (m-TOR); it has also anti-inflammatory effects by regulation of toll-like receptor 4 (TLR4) signaling (21). Previous studies have shown that HFD has a negative effect on AdipoR2 expression in the liver (49). Expression of adiponectin receptors has a close relationship with insulin resistance status and is inversely related to plasma insulin levels. AdipoR expression and insulin can negatively regulate AdipoR2 expression .(50) The present study showed that expressions of AdipoR2 in the liver decreased in the HF group and co-administration of Berberine/ Sitagliptin has stronger effects on expression of AdipoR2 than when administered alone.

Previous reports have shown that high levels of GLUT4 in the liver lower blood sugar and insulin resistance (51, 52). The Sitagliptin-induced up-regulation of GLUT4 in spontaneously hypertensive rats has been partially shown to attribute to an increase in plasma GLP-1 (53), and Sitagliptin can activate the AMPK signaling pathway which leads to an increase in the protein expression of GLUT4 (54). Berberine activates GLUT4-mediated glucose uptake in 3T3-L1 adipocytes (55). Berberine up-regulates GLUT4 via PI3K/AKT activation and MAPK pathway suppression (56). So far no research has been done on the effect of Berberine and Sitagliptin on hepatic GLUT4 expression, and our study showed that co-administration of Berberine/ Sitagliptin increased GLUT4 expression in the liver.

## Conclusion

The results of this study show that co-administration of Berberine/ Sitagliptin reduces insulin resistance, oxidative stress, serum liver enzymes, and serum and liver lipid profiles in male Sprague-Dawley rats fed HF emulsion. Co-administration of Berberine/ Sitagliptin appears to improve insulin resistance, oxidative stress, and dyslipidemia generated by HFD through various synergistic mechanisms. Furthermore, the side effects may be reduced due to halving the dose of drugs in the Berberine/ Sitagliptin group and eventually leads to more effective treatment of NAFLD.
